# 891. Minimum Manufacturing Costs and National Prices for Weight Loss Treatments, as Potential Mitigation for Anti-Retroviral Related Weight Gain in HIV

**DOI:** 10.1093/ofid/ofab466.1086

**Published:** 2021-12-04

**Authors:** Jacob Levi, Junzheng Wang, Francois Venter, Andrew Hill

**Affiliations:** 1 University College London Hospitals, London, UK; 2 Imperial College, London, UK; 3 University of the Witwatersrand, Johannesburg, Gauteng, South Africa; 4 University of Liverpool, London, UK

## Abstract

**Background:**

Weight gain is being observed for a wide range of antiretroviral treatments. Weight gains are higher for people taking first-line integrase inhibitor based treatments, especially those including TAF/FTC. Weight gains are higher for women and people of colour. Clinical obesity increases the risks of cardiovascular disease, diabetes, adverse birth outcomes and could lower survival rates. Anti-obesity treatments are needed to supplement lifestyle interventions and counteract progressive weight gains, but are not routinely provided as part of HIV care.

**Methods:**

Costs of production for FDA-recommended weight loss treatments and anti-diabetic medications (orlistat, naltrexone-bupropion, topiramate, phentermine, semaglutide, liraglutide and metformin) were estimated using an established and published methodology based on costs of active pharmaceutical ingredients (API), extracted from the global shipping records database Panjiva. This was compared with national drug list price data from a range of low, medium, and high-income countries.

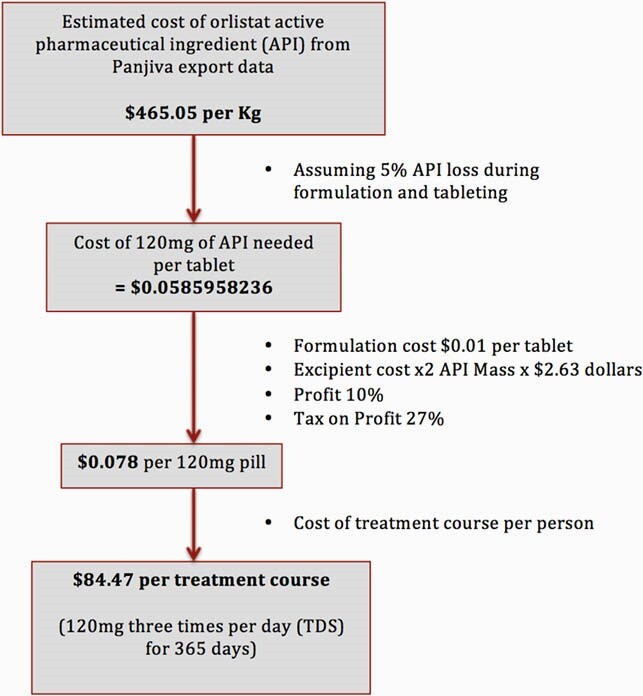

Figure 1. Example of methodology for calculating the estimated minimum cost of production for orlistat

**Results:**

Weight loss and anti-diabetic treatments can be generically manufactured at low per-course costs, e.g. &85 per person per year for oral treatments such as orlistat and &1 per person per month for metformin. However, prices for a year of treatment with orlistat are as high as &1,205 in the USA and as low as &11 in Vietnam. In comparison, a month of ARV treatment costs about &15 via global health institutions like CHAI. Price for injectable (subcutaneous) treatments were higher, ranging from &1,985 for liraglutide in USA to &330 in Morocco, whilst they could potentially be profitably sold for &155 for a 12-week course. No export price data was available for semaglutide. When compared against international list prices, we found wide variations between countries.

Table 1. Summary of drug prices and minimum cost estimates

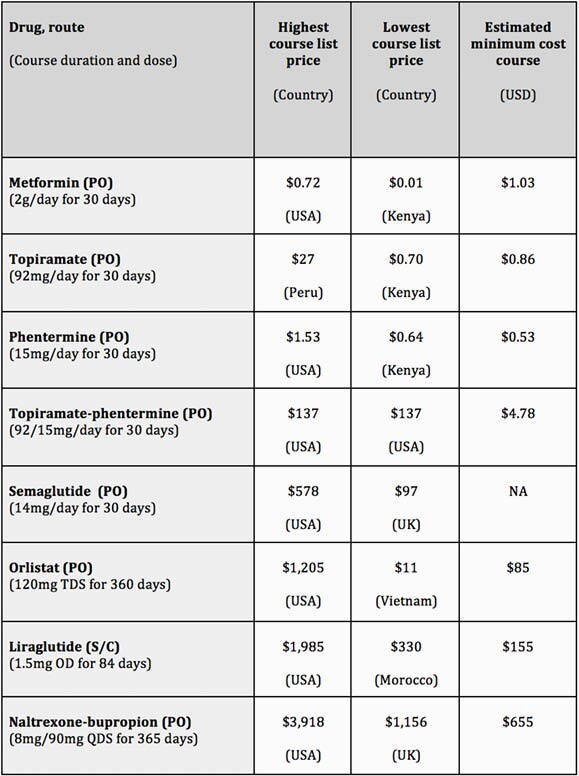

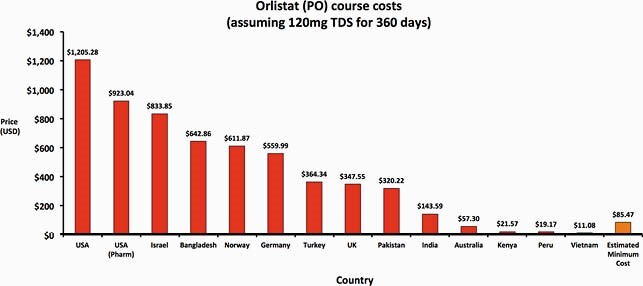

Figure 2. Orlistat course costs in a range of countries, compared with estimated minimum cost

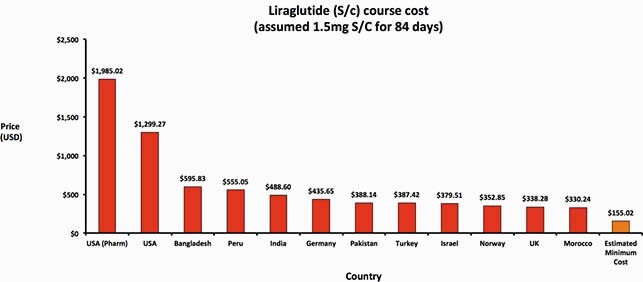

Figure 3. Liraglutide course costs in a range of countries, compared with estimated minimum cost

**Conclusion:**

We show that weight loss treatments can be manufactured and sold profitably for low prices, but have a wide price range between countries. Government and non-governmental healthcare systems should be evaluating weight loss agents for inclusion within ART programmes.

**Disclosures:**

**All Authors**: No reported disclosures

